# COMMUNI.*CARE* (COMMUNIcation and Patient Engagement at Diagnosis of PAncreatic CAncer): Study Protocol

**DOI:** 10.3389/fmed.2020.00134

**Published:** 2020-04-30

**Authors:** Monica Consolandi, Carlo Martini, Michele Reni, Paolo Giorgio Arcidiacono, Massimo Falconi, Guendalina Graffigna, Gabriele Capurso

**Affiliations:** ^1^Faculty of Philosophy, Vita-Salute San Raffaele University, Milan, Italy; ^2^Centre for Philosophy of Social Science, University of Helsinki, Helsinki, Finland; ^3^Department of Medical Oncology, Pancreas Translational and Clinical Research Centre, IRCCS San Raffaele Scientific Institute, Milan, Italy; ^4^Pancreato-Biliary Endoscopy and Endosonography Division, Pancreas Translational and Clinical Research Centre, IRCCS San Raffaele Scientific Institute, Milan, Italy; ^5^Pancreatic Surgery Unit, Pancreas Translational and Clinical Research Centre, IRCCS San Raffaele Scientific Institute, Milan, Italy; ^6^Department of Psychology, EngageMinds Hub Consumer, Food & Health Engagement Research Center, Università Cattolica del Sacro Cuore (Milano), Milan, Italy

**Keywords:** pancreatic cancer, communication, diagnosis, patient engagement, doctor–patient interaction, therapeutic alliance, compliance

## Abstract

**Background:** In many cases of pancreatic adenocarcinoma (PDAC), the diagnosis comes as a surprise to the patient, who often faces a disease that is already at an advanced stage, with poor prognosis. The clinical visit during which the diagnosis is communicated together with the first information regarding the planned treatments is of paramount importance. We hypothesize that the clarity of such information can influence patients' engagement and thus their level of compliance.

**Aims:** This study aims to collect (a) quantitative data on the level of PDAC patient engagement, (b) data on the rate of understanding of the information received from the doctor, and (c) data on level of compliance; the possible associations between these variables will be analyzed.

**Methods:** This is a single-center, observational, cross-sectional cohort study on patients diagnosed with PDAC, approved by the Ethics Committee of the San Raffaele Hospital. As no preliminary data are available on the association between PDAC patients' understanding rate and their level of engagement and of compliance, no power calculation is possible. This is a pilot study, aimed at enrolling at least 45 PDAC patients during a period of 3 months.

**Conclusion:** COMMUNIcation and Patient Engagement at Diagnosis of PAncreatic CAncer (COMMUNI. *CARE*) will be the first study specifically investigating whether there is a relation between PDAC patients' engagement, rate of understanding at the time of diagnosis, and compliance.

## Introduction

### Background

Pancreatic ductal adenocarcinoma (PDAC) is a lethal disease with a standardized incidence rate by age of 4.8 and a standardized mortality rate of 4.4 per 100,000 persons worldwide (1). During the last decades, the prognosis of most common cancer types has dramatically improved, but this is not the case of PDAC. Indeed, despite recent improvements, PDAC 5-years survival rate is only ~8% (2). This is because the majority of PDAC patients show unspecific symptoms, and the diagnosis is usually made only at an advanced stage of the disease (3). In addition, no population screening program is available (4). More than 450,000 patients are diagnosed with PDAC worldwide every year, with a significant burden for the health and socioeconomic systems.

The diagnosis of PDAC in most cases is completely unforeseen by the patient, who faces the disease suddenly, often already at an advanced stage, and without a progressive approach to it. The clinical interview in which the diagnosis and the first information regarding treatment options and prognosis are communicated, therefore, is a key moment.

Doctor–patient communication is considered “time of care,” as stated by the Italian law n.219/2017 (5). In light of that, correct communication is an integral part of the care itself and lays the foundations for the construction of the therapeutic alliance between the treating physician and the patient, which is essential to guarantee compliance with the proposed treatments. There are, however, few studies conducted on doctor–patient communication in cancer patients (6) and none specifically on PDAC patients.

Many recent studies claim that a good level of patient engagement is an essential condition to build a solid therapeutic alliance (7, 8); in this regard, objective measurement scales of the patient engagement level have been developed and have been demonstrated to be useful tools to quantify the patient's involvement within the care process (9, 10). Among them is the Patient Health Engagement Scale (PHE-S®), a recently validated assessment scale of simple and non-invasive use, which requires only the administration of a questionnaire (11).

### Hypothesis

The hypothesis to be tested is that clarity of communication between the treating physician and the patient at the time of diagnosis, and the patient's level of understanding of the information received, influences a patient's engagement and compliance with the care process.

### Aims

This study aims to (1) collect quantitative data about the level of PDAC patients engagement and their rate of understanding of the received information and (2) investigate the association between engagement and rate of understanding and of these two variables with the level of compliance.

## Methods and Analysis

### Study Design

This will be a monocentric cohort study (observational, cross-sectional) on PDAC patients.

### Patients

Consecutive outpatients visited by three expert physicians at the Gastroenterology, Pancreatic Surgery and Oncology Clinics of the Pancreas Translational and Clinical Research Center, IRCCS San Raffaele, Milan (Italy) will be considered includable if they meet the following conditions:

- adult patients (≥18 years)
- of Italian mother tongue
- have a histological diagnosis of PDAC obtained during the 4 weeks prior to the visit
- the visit is their first visit, after completion of diagnostic procedures and is the occasion in which diagnosis and/or treatment strategies are communicated
- give full, written, informed consent.

The following exclusion criteria will be applied:

- PDAC recurrence after previous diagnosis and treatment
- poor performance status [Eastern Cooperative Oncology Group (ECOG) ≥ 3].

### Variables

The following variables will be recorded in a dedicated Case Report Form (CRF): age, sex, region of origin, educational level of the patient, habits such as smoking and alcohol intake as previously reported (12), date of the histological diagnosis of pancreatic adenocarcinoma, its stage, and the proposed treatment plan.

The level of patient engagement will be evaluated through the PHE-s® (see Appendix 1).

The rate of understanding of the information received from the patient by the doctor will be determined during a semistructured interview (see Appendix 2) by comparing the number of information conveyed by the doctor with the number of information received/correctly understood by the patient.

The compliance with treatments will be defined as the rate of treatments received compared to what was proposed and planned by the treating physicians and was considered possible by the medical team in light of patient's conditions and side effects.

### Study Period

The enrolment is planned to start from June 2020 and will last until the planned number of enrolled patients has been met.

### Outcomes

The association between the level of engagement, as evaluated by the PHE-s®, and the patient's rate of understanding during the diagnostic interview, will be assessed to evaluate whether the degree of clarity of the information provided by the doctor influences the level of patient engagement.

The association between the level of patient engagement is calculated with the PHE-s® and patient's estimated rate of understanding according to the interview and the adherence to proposed treatments (compliance).

### Description of the Intervention (Schedule of Visits)

The study requires (i) the completion of the scheduled outpatients visit with a gastroenterologist, surgeon, or oncologist with strong expertise in PDAC, (ii) the filling of the PHE-S® questionnaire by the patient to investigate the level of engagement, and (iii) the completion of a semistructured interview to assess the rate of understanding of the information received from the doctor.

In more detail:

- The treating physician will inform the patient about the study before starting the outpatient visit and communicating information on PDAC diagnosis and planned treatments. The treating physician will also ask permission to the patient regarding the presence during the visit of an external observer (a Ph.D. student in philosophy of language and communication), who will take notes regarding the information provided and will record the audio of the visit if the patient gives further (optional) consent.
- After the visit, the external observer will ask the patient further written informed consent to use the data collected during the interview and to complete the study. Informed consent forms are presented at this stage rather than at a preliminary stage to avoid anticipating the diagnosis of PDAC, which the patient might not have yet received from the doctor.
- The patient will be asked to fill in the PHE-s® questionnaire, which takes ~10 min to be completed. PHE-s® is a standardized and validated tool to measure patients' psychological readiness to engage in patients with different medical disorders.
- The external observer will conduct a semistructured interview of ~30 min to ask the patient questions concerning the clarity of the communication he had received from the doctor and the type of language used, to record the rate of understanding of the information. This phase will ideally take place following the PHE-s® questionnaire, but the patient will be free to postpone it to another moment or day given the possible difficulties for the patient to face an interview immediately following the communication of the diagnosis. The interview will be conducted by an expert observer in the field of language and communication (a Ph.D. student in philosophy of language and communication). The object of the interview is the language used by the treating physician and its clarity for the patient.

All patients will then be treated and followed up normally in the context of the multidisciplinary team of the Pancreas Center that also include the aid of “navigator” and “research” nurses.

### The Medication of the Study

The study is observational, so a preplanned treatment is not considered.

### Statistical Analysis

Categorical variables will be compared using Fisher or chi-square tests and continuous variables using *t* test or Mann–Whitney tests, as appropriate. Subgroups with different levels of engagement will be compared for variables such as sex, age, education, geographic area, stage of disease, type of proposed treatment, and other available data. The PHE-s® questionnaire provides continuous data. The possible correlation of the level of engagement with the clarity of the received information that will be considered as a categorical variable divided in quartiles and with the compliance with treatments will be tested by a Pearson test. Two different univariate and multivariate logistic regression analyses will be run considering patient engagement and compliance as outcome variables and patients' and disease features and the clarity of communication as explanatory variables.

The “enter” method will be employed including all variables that had resulted to be significant at the univariate analysis. Tests of statistical significance and confidence intervals will be two-sided; a *p* < 0.05 will be considered to be statistically significant. A dedicated software (Medcalc 12.1, Belgium) will be employed.

### Power Size Calculation

There have been no previous studies on the association between the patient's understanding rate and the level of engagement and the consequent level of compliance in patients with PDAC. This is, therefore, a pilot study, and power size cannot be calculated. Considering that at S. Raffaele Hospital, some 500 new PDAC patients are seen per year, with the established inclusion criteria, it is considered plausible that 45–60 patients will be enrolled in 3 months.

## Discussion

Active engagement of the patient to the therapeutic program has been shown to be an important factor not only to obtain improved compliance but also to improve the outcomes (13, 14).

Data on the communication between physicians and patients with PDAC are scanty, and patients' engagement has not been systematically evaluated in this context (15).

We hypothesize that the clarity of communication between the treating physician and the patient and the degree of comprehension by the patient, especially during the first phases of the cure, are associated with the level of engagement and consequently with the compliance of patients with PDAC.

If the present pilot study will support this initial hypothesis, further action to improve the clarity of the clinical communication will be promoted.

## Ethics Statement

The study was be performed under the Declaration of Helsinki (2013) as well as the Good Clinical Practice International Ethical and Scientific Quality Standards. The study protocol was approved by the Ethics Committee of the San Raffaele Hospital on June the 14th, 2019, n.52/INT/2019 and is published in ClinicalTrials.gov (NCT04257955). The database does not contain names or identification numbers that may compromise patient anonymity. Participation in the study was voluntary, after signed informed consent. The written informed consent was be obtained by the study collaborators. The results of the study will be disseminated among representatives of the medical community through dedicated medical conferences and published articles.

## Author Contributions

MC and GC conceived the original idea, designed the study, and wrote the paper. GG had developed the PHE-s® utilized in the study and contributed to the final version of the manuscript. CM supervised the theoretical framework of the study. MR, PA, and MF contributed to the design of the study. All authors approved the current manuscript.

## Conflict of Interest

The authors declare that the research was conducted in the absence of any commercial or financial relationships that could be construed as a potential conflict of interest.

## References

[1] International Agency for Research on Cancer: Global Cancer Observatory. Lyon: World Health Organization; c2018 Available online at: https://gco.iarc.fr/ (accessed November 4, 2018).

[2] Siegel RL Miller KD Jemal A. Cancer statistics. CA. (2016) 66:7–30. 10.3322/caac.21332 26742998

[3] Stornello C Archibugi L Stigliano S Vanella G Graglia B Capalbo C Diagnostic delay does not influence survival of pancreatic cancer patients. United Eur Gastroenterol J. (2019) 8:81–90. 10.1177/2050640619879004 PMC7006002 32213057

[4] Signoretti M Bruno MJ Zerboni G Poley JW Delle Fave G Capurso G. Results of surveillance in individuals at high-risk of pancreatic cancer: a systematic review and meta-analysis. United Europ Gastroenterol J. (2018) 6:489–99. 10.1177/2050640617752182 29881603 PMC5987280

[5] Legge 22 dicembre 2017, n.219 Norme in materia di consenso informato e di disposizioni anticipate di trattamento (pubblicata in G.U. Serie Generale 16 gennaio 2018, n.12). Available online at: https://www.gazzettaufficiale.it/eli/id/2018/1/16/18G00006/sg (accessed January 31, 2020).

[6] Adamson M Choi K Notaro S Cotoc C. The doctor-patient relationship and information-seeking behavior: four orientations to cancer communication. J Palliat Care. (2018) 33:79–87. 10.1177/0825859718759881 29514545

[7] D'Angelica M Hirsch K Ross H Passik S Brennan FM. Surgeon-patient communication in the treatment of pancreatic cancer. AMA Arch Surg. (1998) 133:962–6. 10.1001/archsurg.133.9.966 9749848

[8] Geessink NH Ofstad EH Olde Rikkert MGM van Goor H Kasper J Schoon Y. Shared decision-making in older patients with colorectal or pancreatic cancer: determinants of patients' and observers' perceptions. Pat Educ Counsel. (2018) 101:1767–74. 10.1016/j.pec.2018.06.005 29933924

[9] Ibrahim F Sandström P Björnsson B Lindhoff Larsson A Drott J. ‘I want to know why and need to be involved in my own care…’: a qualitative interview study with liver, bile duct or pancreatic cancer patients about their experiences with involvement in care. Support Care Cancer. (2018) 27:7. 10.1007/s00520-018-4548-8 30430301 PMC6541569

[10] Van Rijssen LB Gerritsen A Henselmans I Sprangers MA Jacobs M Bassi C. Core set of patient-reported outcomes in pancreatic cancer (COPRAC). An International Delphi Study Among Patients and Health Care Providers. Ann Surg. (2017) 270:1. 10.1097/SLA.0000000000002633 29261524

[11] Graffigna G Barello S Bonanomi A Lozza E. Measuring patient engagement: development and psychometric properties of the Patient Health Engagement (PHE) Scale. Front Psychol. (2015) 6:274. 10.3389/fpsyg.2015.00274 25870566 PMC4376060

[12] Archibugi L Piciucchi M Stigliano S Valente R Zerboni G Barucca V. Exclusive and combined use of statins and aspirin and the risk of pancreatic cancer: a case-control study. Sci Rep. (2017) 7:1. 10.1038/s41598-017-13430-z 29026148 PMC5638859

[13] Chalmers CC Trust in medicine. J Med Phil. (2002) 27:11–29. 10.1076/jmep.27.1.11.2975 11961684

[14] Bigi S. Key components of effective collaborative goal setting in the chronic care encounter. Commun Med. (2014) 11:1–13. 10.1558/cam.v11i2.21600 26596119

[15] Tang C Draucker C Tejani MA Von Ah D. Patterns of interactions among patients with advanced pancreatic cancer, their caregivers, and healthcare providers during symptom discussions. Support Care Cancer. (2018) 26:3497–506. 10.1007/s00520-018-4202-5 29696423

