# Supplementary material for: COMMUNI.CARE (COMMUNIcation and Patient Engagement at Diagnosis of PAncreatic CAncer): Study Protocol
Source: Front Med (Lausanne). 2020 Apr 30;7:134. doi: 10.3389/fmed.2020.00134 (PMC7203337; doi:10.3389/fmed.2020.00134)
Supplement: Supplementary file 1 [file Data_Sheet_1.docx]

**Appendix 1**

| *Thinking about my health status…* | | | | | | | |
| --- | --- | --- | --- | --- | --- | --- | --- |
|  |  |  |  |  |  |  |  |
| *1* | I feel in blackout  O | O | I feel on the alert  O | O | I am aware  O | O | I feel positive  O |
| *2* | I feel dazed  O | O | I am in alarm  O | O | I am conscious  O | O | I feel serene  O |
| *3* | When I think about my illness I feel overwhelmed by emotions  O | O | I feel anxious every time a new symptom arises  O | O | I have got used to my illness condition  O | O | Despite my illness  I perceive coherence and continuity in my life  O |
| *4* | I feel very discouraged due to my illness  O | O | I feel anxious when I try to manage my illness  O | O | I feel I have adjusted to my illness  O | O | I am generally optimist about my future and my health condition  O |
| *5* | I feel totally oppressed by my illness  O | O | I am upset when a new symptom arises  O | O | I feel I have accepted my illness  O | O | I can give sense to my life despite my illness condition  O |

**Appendix 2**

The investigator draws this report as a memo during phase 2 of the study (the semi-structured interview with the patient after his/her medical interview at the moment of the pancreatic cancer diagnosis). During this phase, the investigator divides the meeting with the patient in two principle moments:

1. the analysis of the PHE-s® together with the patient, who has previously filled in during phase 1;
2. the semi-structured interview to define the patient's level of understanding of the information received during the medical interview.

The report will be audio-recorded.

Some examples of the guide-questions during the semi-structured interview below:

- How do you view your communication with the doctor?
- Has the doctor been thorough in communicating with you?
- Were all of your questions answered, or do you feel you have any doubts left?
- Was the language used understandable?
- Do you agree with the following statement: "trust is the foundation of the doctor-patient relationship"?
- Which are the criteria on the basis of which you chose to trust the doctor? Could you identify any key moments during the interaction?
- When do you think it is the physician who gives his/her trust to the patient? In your specific case, when did it occur?
